# Oral Dexamethasone vs. Oral Prednisone for Children With Acute Asthma Exacerbations: A Systematic Review and Meta-Analysis

**DOI:** 10.3389/fped.2019.00503

**Published:** 2019-12-13

**Authors:** Jienan Wei, Yan Lu, Fang Han, Jing Zhang, Lan Liu, Qingqing Chen

**Affiliations:** ^1^Department of Pediatrics, Shengli Oilfield Central Hospital, Dongying, China; ^2^Department of Hematology, Shengli Oilfield Central Hospital, Dongying, China

**Keywords:** status asthmaticus, prednisolone, dexamethasone, steroids, relapse

## Abstract

**Background:** This systematic review and meta-analysis was conducted to compare relapse rates and adverse effects with oral dexamethasone vs. oral prednisone for acute asthma exacerbations in pediatric patients.

**Methods:** A computerized literature search of PubMed, Embase, Scopus, CENTRAL (Cochrane Central Register of Controlled Trials) and Google scholar databases was carried out till 1st August 2019. Six Randomized controlled trials (RCTs) and 1 quasi-RCT were included. Dosage of dexamethasone and prednisone varied across studies. Studies were grouped based on the follow-up period and duration of dexamethasone administration.

**Results:** There was no significant difference in the relapse rate between dexamethasone and prednisone at 1–5 days (RR 1.46, 95%CI 0.69–3.7, *P* = 0.32; *I*^2^ = 0%) and 10–15 days of follow up (RR 1.16, 95%CI 0.80–1.68, *P* = 0.44; *I*^2^ = 0%). Pooled analysis found no significant difference in relapse rates with 1-day (RR 1.15, 95%CI 0.68–1.95, *P* = 0.60; *I*^2^ = 0%) and 2-day dosage of dexamethasone (RR 1.25, 95%CI 0.82–1.92, *P* = 0.30; *I*^2^ = 0%) compared to prednisone. Hospital readmission rates after initial discharge were not significantly different between the two drugs (RR 1.49, 95%CI 0.56–4.01, *P* = 0.43; *I*^2^ = 0%). Frequency of vomiting at ED (RR 0.21, 95%CI 0.05–0.96, *P* = 0.04; *I*^2^ = 50%) and at home (RR 0.42, 95%CI 0.25–0.69, *P* = 0.0007; *I*^2^ = 0%) was significantly higher with prednisone as compared to dexamethasone.

**Conclusion:** While our results indicate that both dexamethasone and prednisone have similar relapse rates when used for acute asthmatic exacerbations, strong conclusions cannot be drawn due to paucity of large scale RCTs and limited quality of evidence. Dexamethasone is however associated with lower incidence of vomiting as compared to prednisone. Further homogenous RCTs are needed to provide robust evidence on this topic.

## Introduction

Asthma is a common pediatric disease that results in significant limitation of activity and an estimated loss of 14.4 million school days in children (1). The disease is characterized by chronic airway inflammation, airway edema, bronchoconstriction, and airway hyperresponsiveness which results in respiratory symptoms like wheezing, shortness of breath, chest tightness, and cough (2). The intensity of the disease varies with time and episodes of exacerbation frequently require management in the pediatric Emergency Department (ED) (3).

The primary line of treatment in acute exacerbations of asthma is directed at a quick reversal of bronchospasm and reduction of airway inflammation (4). For this purpose, oral steroids are extremely effective for alleviating symptoms in children (5). The 2019 British guidelines on the Management of Asthma recommend inhaled β2 agonists as fist line of treatment for acute asthma exacerbations in children. Early use of oral steroid therapy is also recommended with prednisone being the drug of choice (6). However, despite treatment, around 5–25% of patients relapse and many require admission for management of subsequent exacerbations (7). Relapse after prednisone therapy has been attributed to several factors like the unpleasant bitter taste of the drug, side-effects like vomiting, and its multi-dose regimen of 3–5 days which may reduce patient compliance (8–10).

To improve patient compliance and reduce relapse rates, the role of dexamethasone has been evaluated in many trials (4, 7). Initial studies evaluating a single dose of intramuscular (IM) dexamethasone have found it to be as effective as a 3–5 day regimen of prednisone (11, 12). Subsequently, studies have also compared oral 1 or 2-day therapy of dexamethasone against a 3–5 days regimen of oral prednisone (4, 13). Oral formulations are desirable in children as they are associated with less pain. To date, two meta-analyses have compared oral dexamethasone and prednisone for acute exacerbations of asthma in children, with the last literature search performed in April 2016 (3, 4). Due to the limited number of studies analyzed in these previous reviews, this study aimed to provide an updated Level 1 evidence on relapse rates and adverse effects of oral dexamethasone vs. oral prednisone for acute asthmatic exacerbations in children.

## Materials and Methods

### Criteria for Study Inclusion

The guidelines of the PRISMA statement (Preferred Reporting Items for Systematic Reviews and Meta-analyses) (14) and the Cochrane Handbook for Systematic Reviews of Intervention were followed during the conduct of this review (15). Only quasi-randomized controlled trials (RCTs) and RCTs were included in our study. Utilizing the PICOS (Population, Intervention, Comparison, Outcome, and Study design) outline for selecting studies, we included trials conducted in pediatric patients (<18 years) with acute asthmatic exacerbation treated in either an ambulatory or ED setting (*Population*); comparing oral dexamethasone (*Intervention*) with oral prednisone (*Comparison*); and assessing relapse rates and adverse events (*Outcomes*). Studies including adult asthma patients and utilizing the parenteral route of administration of dexamethasone or prednisone were excluded. We also excluded non-randomized studies, retrospective studies, case-series, and non-English language studies.

### Search Strategy

A computerized literature search of PubMed, Embase, Scopus, CENTRAL (Cochrane Central Register of Controlled Trials) and Google scholar databases was carried out. The last literature search was conducted on 1st August 2019. Two independent reviewers performed the electronic search using the following key-words: “dexamethasone,” “prednisone,” “prednisolone,” “decadron,” “asthma,” “status asthmaticus,” “children,” “randomized controlled trials,” and “relapse.” We also performed a manual search of references of included studies and review articles on the subject for additional inclusion. After assessing the studies by their titles and abstracts, full-texts of selected articles were retrieved. Both the reviewers assessed individual studies based on inclusion criteria. Disagreements, if any, were resolved by mutual agreement.

### Data Extraction

Using an abstraction form, two reviewers retrieved data from selected studies. The following details were sourced: Authors, publication year, sample size, inclusion/exclusion criteria, baseline characteristics, dexamethasone and prednisone protocol, additional drug therapy, outcomes, and adverse events. The primary outcome was the relapse rate defined by an unscheduled visit to the ED or clinic. Secondary outcomes were hospital readmission after discharge and incidence of vomiting at ED or home.

### Risk of Bias Assessment and Statistical Analysis

The “Cochrane Collaboration risk assessment tool for RCTs” was selected for quality assessment of the included trials (16). Every study was evaluated for the following variables: random sequence generation, allocation concealment, blinding of participants and personnel, blinding of outcome assessment, incomplete outcome data, selective reporting, and other biases. We rated studies on each variable as low risk, high risk, or unclear risk of bias.

Anticipating heterogeneity amongst studies, a random-effects model was used to calculate the pooled effect size. Categorical data were summarized using the Mantel-Haenszel Risk Ratio (RR) with 95% confidence intervals (CI). Heterogeneity was calculated using the *I*^2^ statistic. *I*^2^ values of 25–50% represented low, values of 50–75% medium and more than 75% represented substantial heterogeneity. A sensitivity analysis was carried out to assess the influence of each study on the pooled effect size. Sub-group analysis was conducted for relapse rates based on follow-up period (1–5 days or 10–15 days) and dosage of dexamethasone. Using the method described by Muncer et al. (17), power of included studies for the every variable was calculated. The software “Review Manager” (RevMan, version 5.3; Nordic Cochrane Center [Cochrane Collaboration], Copenhagen, Denmark; 2014) was used for the meta-analysis. Gpower software was used to calculate the power of studies.

## Results

**Figure 1:**
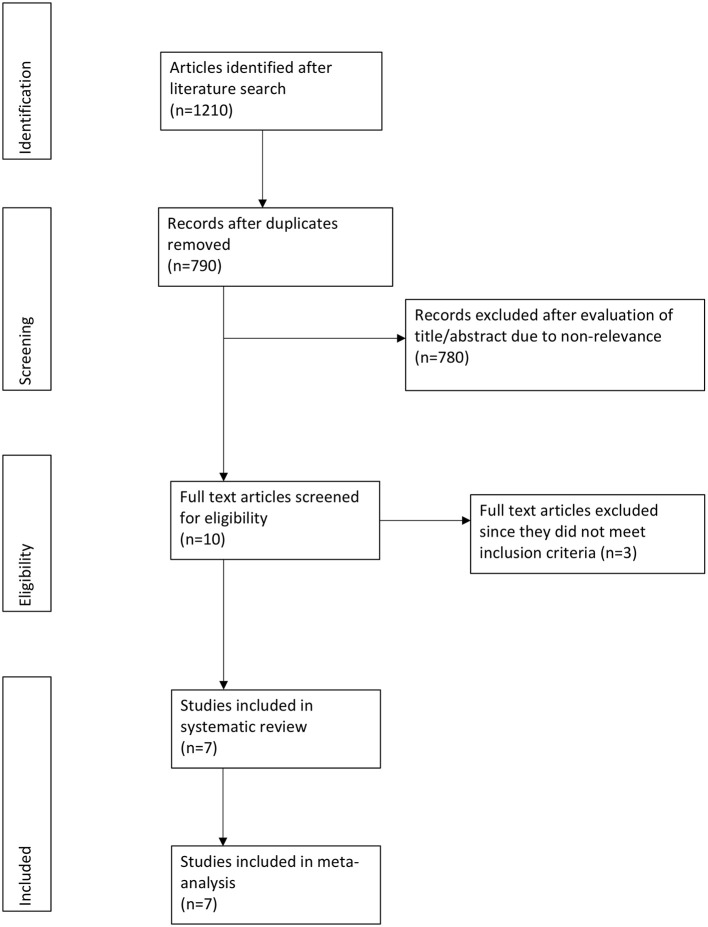
Study flow chart.

Out of the 790 potentially relevant articles, 10 were selected for full-text analysis (Figure 1). Three studies evaluated intramuscular dexamethasone vs. prednisone and hence were excluded (9, 11, 12). A total of seven unique articles were included in this systematic review and meta-analysis (1, 7, 13, 18–21).

Details of individual studies are presented in Table 1. There were six RCTs (1, 13, 18–21) and one quasi-RCT (7). All trials included pediatric patients, however, the age group varied across studies. All studies were performed in the ED with varying sample sizes (23–288 patients). There were some differences in the inclusion/exclusion criteria amongst included studies with regards to severity of asthma. One study excluded patients with severe asthma exacerbation (18). One included patients with moderate severity of exacerbation only (1) while another study included patients with moderate to severe exacerbations (19). Asthma severity was measured on different scales across trials. With an exception of one study (20), there was no statistical significant difference between asthma severity scores of the two study groups. Dexamethasone was administered as a 1-day (1, 13, 18) or 2-days therapy (7, 20, 21). In one trial, patients were randomized into three groups of 1-day dexamethasone, 2-days dexamethasone, and prednisone (19). Data of both dexamethasone groups were compared separately with prednisone in our meta-analysis. The dosage of dexamethasone in the included studies was 0.3–0.6 mg/kg with the maximum dose varying from 12 to 18 mg. In comparison, prednisone was administered for 3–5 days at 1–2 mg/kg for a maximum of 30–80 mg across trials. Majority studies had an institutional asthma management protocol wherein additional drugs were given to all patients of the trial. Inhaled or nebulized salbutamol, albuterol, and ipratropium bromide were commonly administered in the included studies. The follow-up period ranged from 1 to 15 days.

**Table 1:** Details of included studies.

**References**	**Sample size**		**Age group**	**Inclusion criteria**	**Exclusion criteria**	**Asthma severity**		**Dexamethasone protocol**	**Prednisone protocol**	**Allowed medication**	**Follow-up**
	**Dexame-thasone**	**Predn-isone**				**Dexame-thasone**	**Predn-isone**				
Qureshi et al. (7)	272	261	2–18 y	1. Known history of asthma (2 or more episodes of wheezing treated with β-adrenergic agonists ± steroids) 2. Presented to the ED with an acute exacerbation 3. Required at least 2 albuterol nebulizer treatments in the ED	1. Steroid use in past 4 weeks 2. History of intubation 3. Varicella exposure <3 weeks 4. Concurrent stridor, foreign body aspiration 5. Significant respiratory distress necessitating airway intervention 6. Chronic lung or cardiac disease	Mild^a^: 22% Moderate: 56% Severe: 22%	Mild^a^: 23% Moderate: 56% Severe: 21%	Dexamethasone 0.6 mg/kg (max 16 mg) PO once daily × 2 d	Prednisone 2 mg/kg (max 60 mg) PO × 1 dose, then 1 mg/kg (max 60 mg) PO daily × 4 d	Nebulized albuterol and ipratropium for all patients in ED. Albuterol inhalations on a 4–6 h basis for the first 2 d after discharge, then as needed.	11–14 d
Altamimi et al. (18)	67	67	2–16 y	1. Presenting to the ED with an acute exacerbation of mild to moderate asthma (PIS <9 or PEFR of ≥60% of predicted value for height) 2. History of at least 1 prior episode suggestive of “asthma-like” acute shortness of breath or wheezing that was treated with salbutamol	1. Severe asthma on presentation (PEFR <60%, PIS ≥ 10) 2. Complete recovery after the first salbutamol therapy 3. Steroid use in past 2 weeks 4. History of severe asthma exacerbation 5. History of intubation or intensive care unit admission for asthma 6. Chronic lung disease, heart disease, neurological disorder, psychiatric disease 7. History of acute allergic reaction, active chickenpox, or herpes simplex infections	PIS: 6 ± 1.74	PIS: 5.7 ± 1.97	Dexamethasone 0.6 mg/kg (max 18 mg) PO × 1 dose, placebo PO twice daily × 5 d	Prednisone 1 mg/kg (max 30 mg) PO × 1 dose, then 1 mg/kg PO twice daily × 5 d	Salbutamol	5 d
Greenberg et al. (20)	51	38	2–18 y	1. Known history of asthma (2 or more episodes of wheezing treated with β-adrenergic agonists) 2. Presented to the ED with an acute exacerbation	1. Steroid use in past 1 month 2. History of intubation 3. Varicella exposure <3 weeks 4. Foreign body aspiration or chronic lung disease 5. Significant respiratory distress necessitating airway intervention 6. ≥2 episodes of emesis after steroid administration in the ED 7. Chronic heart, liver, or kidney disease	PAS^b^: 8 (range: 5–14)	PAS^b^: 6 (range: 5–12)	Dexamethasone 0.6 mg/kg (max 16 mg) PO once daily × 2 d	Prednisone 2 mg/kg (max 80 mg) PO × 1 dose, then 1 mg/kg (max 30 mg) PO twice daily × 4 d	Nebulized albuterol and ipratropium for all patients in ED. Albuterol every 4 h for 24 h after discharge, then as needed.	10 d
Cronin et al. (13)	123	122	2–16 y	1. Known history of asthma (1 or more episodes of wheezing treated with β-adrenergic agonists) 2. Presented to the ED with an acute exacerbation	1. Critical or life-threatening asthma exacerbation^b^ 2. Active varicella or herpes simplex infection, concurrent infection with respiratory syncytial virus, temperature >39.5°C 3. Steroid use in past 4 weeks 4. Concurrent stridor, galactose intolerance, the Lapp-lactase deficiency or glucose-galactose malabsorption 5. History of tuberculosis exposure, or significant comorbid disease	PRAM: 4.38 ± 2.53	PRAM: 4.51 ± 2.35	Dexamethasone 0.3 mg/kg (max 12 mg) PO once daily × 1 d	Prednisone 1 mg/kg (max 40 mg) PO daily × 3 d	Inhaled beta-2-agonist and corticosteroids	14 d
Paniagua et al. (21)	288	283	1–14 y	1. Known history of asthma (1 or more episodes of wheezing treated with β-adrenergic agonists) or first episode of wheezing in children >2 years and history of atopy 2. Presented to the ED with an acute exacerbation	1. Critical or life-threatening asthma exacerbation 2. Steroid use in past 4 weeks 3. Presentation with respiratory failure that needed further support such as intravenous steroids, intravenous magnesium sulfate, and/or high-flow oxygen and admission to the pediatric intensive care unit	PS^c^: 4.5 ± 1.4 O_2_ saturation: 92.1 ± 19.2	PS: 5.1 ± 1 O_2_ saturation: 93.9 ± 2.7	Dexamethasone 0.6 mg/kg (max 12 mg) PO once daily × 2 d	Prednisone 1.5 mg/kg (max 60 mg) PO × 1 dose, then 1 mg/kg (max 60 mg) PO twice daily × 4 d	Nebulized albuterol and/or ipratropium for all patients in ED. Albuterol every 2–6 h for 24 h after discharge, then as recommended by pediatrician	7 d, 15 d
Elkharwili et al. (19)	G1:29 G2: 29	23	2–11 y	1. Known history of asthma 2. Presented to the ED with an acute exacerbation 3. Moderate to severe asthma failing to respond with short acting β-agonists only^d^	1. History of intubation for previous asthma exacerbations. 2. Active varicella or herpes simplex infection in <3 weeks 3. Concurrent infection with respiratory syncytial virus. 4. Steroid use in past 4 weeks 5. Concurrent stridor, known patients with tuberculosis and the presence of other significant comorbidities, such as cardiac, immune, liver, endocrine, neurological, and psychiatric disorders.	G1: PEFR- 53 ± 13.17 G2: PEFR-52.8 ± 14.14	PEFR-54.25 ± 13.2	G1: Dexamethasone 0.3 mg/kg (max 12 mg) PO once daily × 1 d G2: Dexamethasone 0.6 mg/kg (max 16 mg) PO in three divided doses × 2 d	Prednisone 1.5 mg/kg (max 60 mg) PO in three divided doses × 5 d	NR	5 d
Prasanna- venkatesh et al. (1)	30	30	2–12 y	1. Known history of asthma 2. Presented with acute exacerbation of moderate severity (PRAM score of 5–8)	1. Lower respiratory infection, other serious infections or organic diseases 2. Steroid use in past 1 week	PRAM: 5.83 ± 0.83	PRAM: 6.13 ± 0.82	Dexamethasone 0.3 mg/kg (max 12 mg) PO once daily × 1 d	Prednisone 1 mg/kg (max 40 mg) PO once daily × 3 d	Levosalbutamol and ipratropium for all patients in ED	24 h

*G1, Group 1; G2, Group 2; y, years; max, maximum; PO, per-oral; PIS, Pulmonary Index Score; PEFR, Peak expiratory flow rate; PAS, Pediatric asthma score; PRAM, Pediatric Respiratory Assessment Measure; PS, Pulmonary Score; d, day; ED; Emergency Department; h, hours; NR, Not Reported*.

a *Exacerbation was classified as mild, moderate, or severe based either on the percentage of a child's predicted PEFR or an asthma score. Asthma episode was classified as “mild” if the PEFR was >70% of the predicted value or the asthma score was 5–7, “moderate” if the PEFR was 50% to 70% of the predicted value or the asthma score was 8–11, or “severe” if the PEFR was <50% of the predicted value or the asthma score was 12–15*.

b *A critical or life-threatening asthma exacerbation was defined as patients displaying 1 or more of the following clinical features: confused or drowsy, maximal accessory muscle use or recession, poor respiratory effort (including bradypnea), exhaustion, silent chest, cyanosis, SaO_2_ <90% in air, marked tachycardia, unable to verbalize normally (i.e., different from baseline verbal ability), and pneumothorax*.

c *Using the pulmonary score (PS) and oxygen saturation, patients were classified into 1 of 3 levels: mild (PS ≤ 3, O_2_ saturation >94%), moderate (PS 4–6, O_2_ saturation 91–94%), or severe (PS > 6, O_2_ saturation <91%)*.

d *Criteria for moderate to severe asthma not defined*.

**Figure 2:**
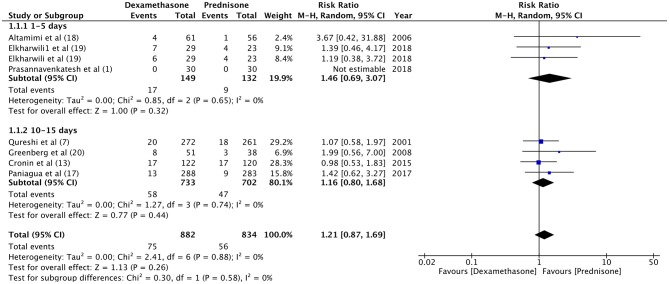
Forrest plot for dexamethasone vs. prednisone for relapse rates with sub-group analysis based on follow-up period.

**Figure 3:**
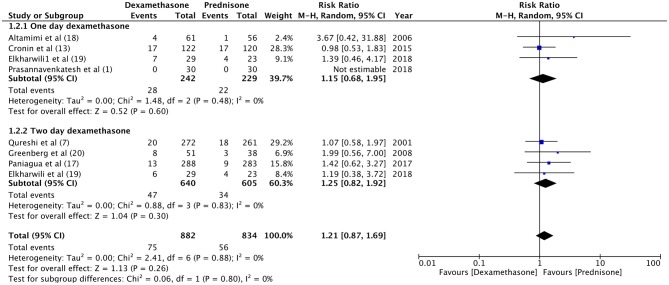
Forrest plot for dexamethasone vs. prednisone for relapse rates with sub-group analysis based on duration of dexamethasone dosage.

For the meta-analysis, two sub-groups were created based on the follow-up period of relapse rates (1–5 days and 10–15 days). There was no significant difference in the relapse rate between dexamethasone and prednisone at 1–5 days (RR 1.46, 95%CI 0.69–3.7, *P* = 0.32; *I*^2^ = 0%) and 10–15 days of follow-up (RR 1.16, 95%CI 0.80–1.68, *P* = 0.44; *I*^2^ = 0%) (Figure 2). With an overall relapse rate of 8.5% with dexamethasone and 6.7% with prednisone, the meta-analysis found no difference between the two groups (RR 1.21, 95%CI 0.87–1.69, *P* = 0.26; *I*^2^ = 0%). Sub-group analysis was carried out for 1-day and 2-days dosage of dexamethasone vs. prednisone (Figure 3). Pooled analysis found no significant difference in relapse rates with 1-day (RR 1.15, 95%CI 0.68–1.95, *P* = 0.60; *I*^2^ = 0%) and 2-days dosage of dexamethasone (RR 1.25, 95%CI 0.82–1.92, *P* = 0.30; *I*^2^ = 0%) compared to prednisone.

**Figure 4:**
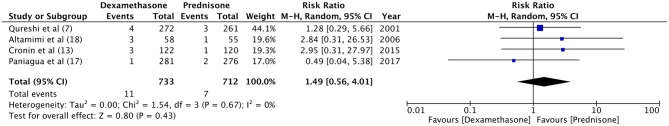
Forrest plot for dexamethasone vs. prednisone for hospital readmission.

**Figure 5:**
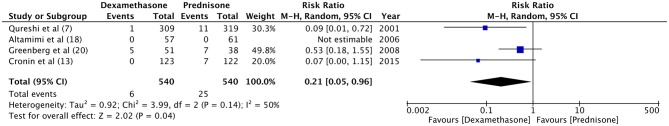
Forrest plot for dexamethasone vs. prednisone for vomiting in Emergency Department.

**Figure 6:**
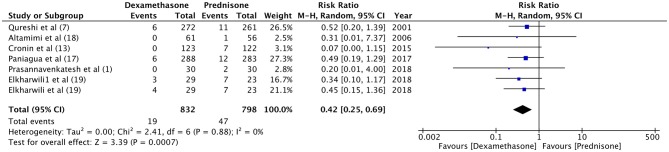
Forrest plot for dexamethasone vs. prednisone for vomiting at home.

Hospital readmission after initial discharge was evaluated by four trials (7, 13, 18, 21). With a re-admission rate of 1.5% in the dexamethasone group and 0.9% in the prednisone group, our meta-analysis found no significant difference between the two drugs (RR 1.49, 95%CI 0.56–4.01, *P* = 0.43; *I*^2^ = 0%) (Figure 4). Data on the incidence of vomiting in ED were retrieved from four studies (7, 13, 18, 20). Patients receiving dexamethasone vomited less frequently as compared to prednisone (RR 0.21, 95%CI 0.05–0.96, *P* = 0.04; *I*^2^ = 50%) (Figure 5). The frequency of vomiting at home was significantly higher with prednisone (5.88%) as compared to dexamethasone (2.28%) (RR 0.42, 95%CI 0.25–0.69, *P* = 0.0007; *I*^2^ = 0%) (Figure 6).

### Sensitivity Analysis and Power of Studies

**Figure 7:**
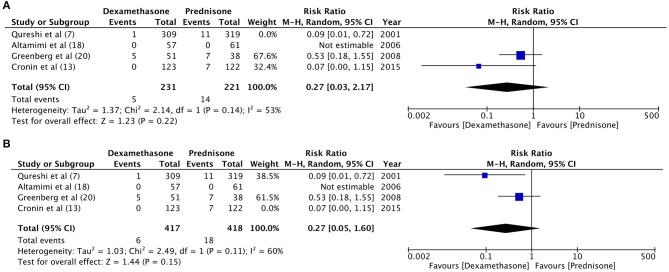
Sensitivity analysis for “vomiting at home” with elimination of trials of **(A)** Qureshi et al. (7) **(B)** Cronin et al. (13).

There was no change in direction of effect size on sensitivity analysis for the variables: relapse rate, hospital readmission, and vomiting at home. However, when the results of Qureshi et al. (7) and Cronin et al. (13) were eliminated individually the pooled analysis demonstrated no difference between dexamethasone and prednisone for “vomiting in ED” [Qureshi et al. (7) eliminated: RR 0.27, 95%CI 0.03–2.17, *P* = 0.22; Cronin et al. (13) eliminated: RR 0.27, 95%CI 0.05–1.60, *P* = 0.15] (Figure 7).

The calculated power of individual studies based on the weighted mean effect size at α = 0.05 is presented in Table 2 (17). For the outcome variables, relapse rate and hospital readmission rate, all studies were underpowered. The weighted mean effect size for relapse rate was 0.0316 and for hospital readmission rate was 0.0132. Based on these values, a total sample size of 8,162 participants and 45,041 participants are required for detecting difference in relapse rate and hospital readmission rate, respectively (α = 0.05, power = 80%). The power of our meta-analysis for detecting significant difference in relapse rate was 25.8% and for hospital readmission was 7.92%. For the variables, vomiting at ED and vomiting at home, the weighted mean effect sizes were 0.1156 and 0.0944, respectively. The power of our meta-analysis at α = 0.05 was 96.9% for both these variables.

**Table 2:** Power analysis of included studies for different outcome variables.

	**Power of the study**			
	**Relapse rate**	**Hospital readmission**	**Vomiting in ED**	**Vomiting at home**
Qureshi et al. (7)	0.1102	0.0603	0.825	0.5854
Altamimi et al. (18)	0.0627	0.0521	0.2386	0.1732
Greenberg et al. (20)	0.0596	-	0.1906	-
Cronin et al. (13)	0.0768	0.0546	0.4384	0.3131
Paniagua et al. (21)	0.1146	0.0608	-	0.6150
Elkharwili et al. (19)	0.0555	-	-	0.1024
Prasannavenkatesh et al. (1)	0.0564	-	-	0.1110

*ED, Emergency Department*.

### Risk of Bias Assessment

**Figure 8:**
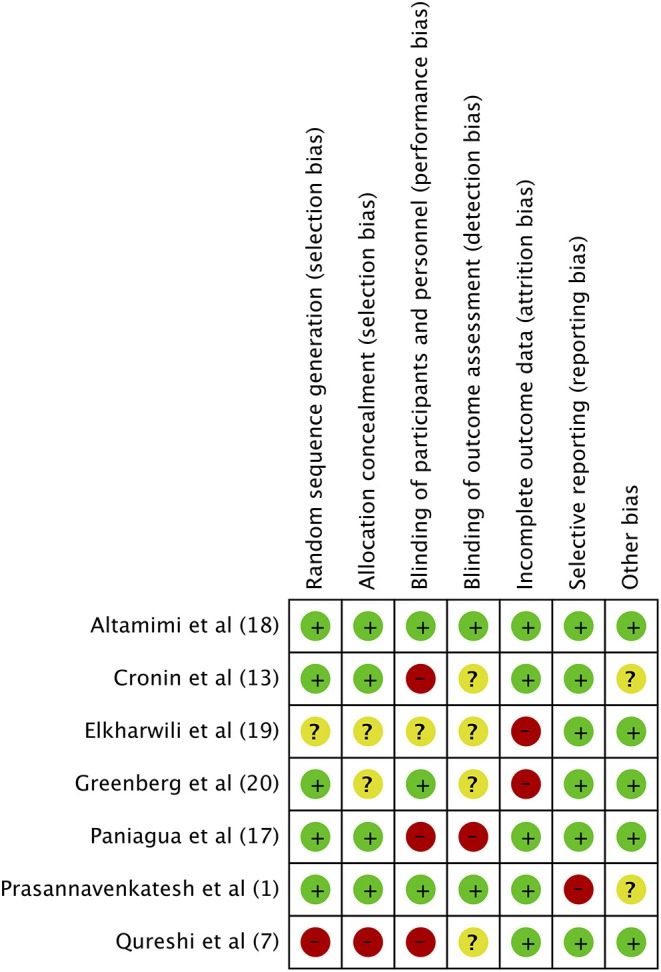
Risk of Bias assessment. Green, low risk of bias; Yellow, unclear risk of bias; Red, high risk of bias.

The authors' judgment of the risk of bias is presented in Figure 8. Randomization was adequately described in five studies (1, 13, 18, 20, 21). An appropriate method of allocation concealment was utilized in four trials (1, 13, 18, 21). Only three studies (1, 18, 20) provided sufficient information on blinding of participants and personnel while only two trials (1, 18) reported blinding of outcome assessment. Attrition bias was found to be high in two studies (19, 20).

## Discussion

Management of acute asthma exacerbations in children not only depends on the therapy provided in the ED but also on strict adherence to medications prescribed on discharge. In a study by Cooper and Hickson (22), <50% of pediatric asthmatic patients reportedly filled their oral corticosteroid prescription following discharge from ED or in-patient department. On the other hand, Butler et al. (8) while reporting a high prescription filling rate of 98.7%, found an overall adherence rate of only 64% to the prescribed length of corticosteroid therapy. Non-compliance to medications on discharge has been attributed to several factors like inadequate funds or lack of insurance, insufficient knowledge on the necessity of treatment, fear of side-effects and prolonged course of treatment (3, 8, 11).

Dexamethasone, a long-acting corticosteroid, has been studied as an alternative to prednisone to allow a shorter course of treatment in asthmatic patients (4). While inhaled and single-dose IM dexamethasone may be used as a substitute to prednisone, oral formulation is preferable in managing children (9, 23). Dexamethasone is well-absorbed orally achieving a relative bioavailability of 70% of an IM formulation (7). Studies conducted on adult asthmatic patients have found no difference in relapse rates with 2-days oral dexamethasone and 5-days prednisone (24, 25). Rehrer et al. (26) while reporting slightly higher relapse rates with 1-day dexamethasone (12.1%) as compared to 5-days prednisone (9.8%) in adult asthma exacerbations concluded that better compliance and practicality of dexamethasone therapy may still substantiate its use.

In our study, while comparing the use of oral dexamethasone and prednisone in pediatric asthma exacerbations, we found no difference in relapse rates between a short-course of dexamethasone as compared to the standard 3–5 days therapy of prednisone. The hospital readmission rates after initial discharge were slightly higher with dexamethasone as compared to prednisone (1.5 vs. 0.9%, *p* = 0.26), however, our results were grossly underpowered. The results of our study are similar to the previous meta-analyses on this subject. Keeney et al. (4) in their review of pediatric asthma exacerbations found no significant difference in the risk of relapse between dexamethasone and prednisone at 5-days (RR 0.90, 95% CI 0.46–1.78) and 10–14 days (RR 1.14 95% CI 0.77–1.67). However, results from both IM and oral dexamethasone trials were pooled in their analysis. Normansell et al. (3) in a meta-analysis of four RCTs, also reported no difference in relapse rates between the two corticosteroids (RR 0.85, 95%CI 0.54–1.34, *P* = 0.48). In comparison, while our updated review was able to include three more RCTs, we also conducted a power analysis to identify if the included studies and our meta-analysis was adequately powered to detect significant difference in outcome variables. It is important to note that despite pooling of data from seven RCTs, our meta-analysis was underpowered to detect significant difference in relapse rates and hospital readmission rates between dexamethasone and prednisone.

In a sub-group analysis, we also compared 1-day and a 2-days course of dexamethasone with 3–5 day therapy of prednisone. However, with just one trial reporting a three-way comparison of 1-day and 2-days dexamethasone with prednisone (19), combined with limited power of our meta-analysis, conclusion cannot be drawn till further studies are carried out to explore the differences between 1 and 2-days dexamethasone protocol. It is also important to note that relapse rates and hospital readmission rates may be influenced by factors like clinical decisions, hospital criteria for admission and accessibility to healthcare facilities (27). The criteria for relapse rate are also broad varying from the visit of the child to a family doctor for continued wheezing and cough to a severe relapse requiring in-patient management (21). Therefore, objective measures of reduction in asthma severity and assessment of persistent symptoms may better evaluate the differences in the two steroid treatment protocols. Elkharwili et al. (19) in their trial found no difference in Pediatric Respiratory Assessment Measure (PRAM), Modified Pulmonary Index Score, and pulmonary function tests with dexamethasone and prednisone at 5 days of follow-up. Paniagua et al. (21) evaluated the persistence of symptoms and quality of life scores with dexamethasone and prednisone and reported no significant difference between the two groups. PRAM scores were measured at day 4 of treatment by Cronin et al. (13) and were not found to be different with dexamethasone or prednisone. Altamimi et al. (18) recorded the number of days required for Patient Self-Assessment Score to return to baseline following steroid therapy. With a mean of 5.21 days with dexamethasone and 5.22 days with prednisone, the authors reported no difference between the two drugs. While individually all included studies reported dexamethasone to be as efficacious as prednisone, methodological heterogeneity of outcome measures precluded a meta-analysis of such variables in our study.

Despite intra-venous dexamethasone and prednisone demonstrating similar efficacy for preventing nausea and vomiting after chemotherapy (28), the unpleasant taste of oral prednisone frequently results in vomiting especially in children (29). While the difference of taste between dexamethasone and prednisone has reportedly not been a hindrance in treatment adherence (21), vomiting may affect treatment compliance in pediatric patients (10). Hames et al. (29) in a comparative study have demonstrated a statistically significant difference between the taste of dexamethasone and prednisolone in asthmatic children, with dexamethasone being the steroid of choice. In our meta-analysis, vomiting at ED and home was found to be significantly higher with prednisone as compared to dexamethasone. Similar results have been reported in the meta-analysis of Keeney et al. (4).

The strengths and limitations of our study need to be elaborated. Firstly, in our review three more RCTs were added since the last published meta-analysis, thereby providing an updated evidence. Secondly, a sensitivity analysis was carried out to assess influence of individual studies on the overall results. Lastly, power analysis was also carried out to provide a guide to readers on the validity of the calculated results. The results of our review, however, should be interpreted with caution due to the following limitations. Foremost, blinding of personnel & participants and outcome assessors were not carried out in the majority of included studies. Additionally, lack of adequate randomization and allocation concealment, as well as attrition bias in some studies, could have influenced the overall results. Secondly, there was considerable methodological heterogeneity amongst the seven trials especially concerning drug dose, duration of therapy, utilization of additional drugs, follow-up protocol, etc. Thirdly, inclusion criteria varied amongst studies, with the trial of Paniagua et al. (21) including participants of <2 years. Asthma is usually not diagnosed at such a young age due to the prevalence of bronchiolitis in this age-group (13). Lastly, our meta-analysis was not adequately powered to detect differences in relapse rates and hospital readmission rates, as out of the seven included trials, three studies (1, 19, 20) were of small sample size recruiting only 23–51 patients per group. In our power analysis, all included studies were found to be underpowered for detection of significant difference in the primary outcome variable.

## Conclusion

To conclude, despite our results indicating similar relapse rates and hospital re-admission rates with dexamethasone and prednisone when used for acute asthmatic exacerbations in children, strong conclusions cannot be drawn due to paucity of large scale RCTs and limited quality of evidence. It is also not known if both drugs are equally efficacious in reducing asthma severity. Our results however indicate that, vomiting is significantly less with dexamethasone as compared to prednisone. Further large-scale homogenous RCTs comparing the two drugs are warranted to establish guidelines for the use of oral steroid therapy in acute asthma exacerbations in children.

## Data Availability Statement

All datasets analyzed for this study are included in the manuscript/supplementary files.

## Author Contributions

JW and QC conceived and designed the study. YL, FH, JZ, and LL collected the data and performed the literature search. JW was involved in the writing of the manuscript. All authors have read and approved the final manuscript.

### Conflict of Interest

The authors declare that the research was conducted in the absence of any commercial or financial relationships that could be construed as a potential conflict of interest.

## References

[1] Prasannavenkatesh K Gunasekaran D Pandi K Srinivasaraghavan R Anandhi C Soundararajan P Single-dose oral dexamethasone compared with three day course of oral prednisolone in children with moderate exacerbation of asthma-a pilot double-blinded randomised controlled trial. J Clin Diag Res. (2018) 12:SC01–3. 10.7860/JCDR/2018/30505.11171

[2] Scarfone RJ Friedlaender E. Corticosteroids in acute asthma: past, present, and future. Pediatr Emerg Care. (2003) 19:355–61. 10.1097/01.pec.0000092585.40174.f6 14578839

[3] Normansell R Kew KM Mansour G. Different oral corticosteroid regimens for acute asthma. Cochrane Database Syst Rev. (2016) CD011801. 10.1002/14651858.CD011801.pub2 27176676 PMC8504986

[4] Keeney GE Gray MP Morrison AK Levas MN Kessler EA Hill GD. Dexamethasone for acute asthma exacerbations in children: a meta-analysis. Pediatrics. (2014) 133:493–9. 10.1542/peds.2013-2273 24515516 PMC3934336

[5] Schwarz ES Cohn BG. Is dexamethasone as effective as prednisone or prednisolone in the management of pediatric asthma exacerbations? Ann Emerg Med. (2015) 65:81–2. 10.1016/j.annemergmed.2014.05.023 24954577

[6] BTS/SIGN. British Guideline on the Management of Asthma. (2019). Available online at: https://www.brit-thoracic.org.uk/quality-improvement/guidelines/asthma/ (accessed 10 August 2019).

[7] Qureshi F Zaritsky A Poirier MP. Comparative efficacy of oral dexamethasone versus oral prednisone in acute pediatric asthma. J Pediatr. (2001) 139:20–6. 10.1067/mpd.2001.115021 11445789

[8] Butler K Cooper WO Adherence of pediatric asthma patients with oral corticosteroid prescriptions following pediatric emergency department visit or hospitalization. Pediatr Emerg Care. (2004) 20:730–5. 10.1097/01.pec.0000144914.78124.6f 15502653

[9] Gordon S Tompkins T Dayan PS. Randomized trial of single-dose intramuscular dexamethasone compared with prednisolone for children with acute asthma. Pediatr Emerg Care. (2007) 23:521–7. 10.1097/PEC.0b013e318128f821 17726409

[10] Lucas-Bouwman ME Roorda RJ Jansman FG Brand PL Short report: crushed prednisolone tablets or oral solution for acute asthma? Arch Dis Child. (2001) 84:347–8. 10.1136/adc.84.4.347 11259239 PMC1718704

[11] Gries DM Moffitt DR Pulos E Carter ER. A single dose of intramuscularly administered dexamethasone acetate is as effective as oral prednisone to treat asthma exacerbations in young children. J Pediatr. (2000) 136:298–303. 10.1067/mpd.2000.103353 10700684

[12] Klig JE Hodge D Rutherford MW. Symptomatic improvement following emergency department management of asthma: a pilot study of intramuscular dexamethasone versus oral prednisone. J Asthma. (1997) 34:419–25. 10.3109/02770909709055384 9350159

[13] Cronin JJ McCoy S Kennedy U An Fhailí SN Wakai A Hayden J. A randomized trial of single-dose oral dexamethasone versus multidose prednisolone for acute exacerbations of asthma in children who attend the emergency department. Ann Emerg Med. (2016) 67:593–601.e3. 10.1016/j.annemergmed.2015.08.001 26460983

[14] Moher D Liberati A Tetzlaff J Altman DG PRISMA Group Preferred reporting items for systematic reviews and meta-analyses: the PRISMA statement. PLoS Med. (2009) 6:e1000097 10.1371/journal.pmed.1000097 19621072 PMC2707599

[15] Higgins J Green S Cochrane Handbook for Systemic Reviews of Interventions Version 5. The Cochrane Collaboration (2011). Available online at: https://training.cochrane.org/handbook/archive/v5.1/ (accessed August 1, 2019).

[16] Higgins J Altman D Sterne J Cochrane statistical methods group and the cochrane bias methods group. Chapter 8: assessing risk of bias in included studies. In: Cochrane Handbook for Systemic Reviews of Interventions. The Cochrane Collaboration. (2011). Available online at: http://www.cochrane-handbook.org (accessed August 1, 2019).

[17] Muncer S Taylor S Craigie M. Power dressing and meta-analysis: incorporating power analysis into meta-analysis. J Adv Nurs. (2002) 38:274–80. 10.1046/j.1365-2648.2002.02177.x 11972663

[18] Altamimi S Robertson G Jastaniah W Davey A Dehghani N Chen R. Single-dose oral dexamethasone in the emergency management of children with Exacerbations of Mild to Moderate Asthma. Pediatr Emerg Care. (2006) 22:786–93. 10.1097/01.pec.0000248683.09895.08 17198210

[19] Elkharwili DA Ibrahim OM Elazab GA Two regimens of dexamethasone versus prednisolone for acute exacerbations in asthmatic Egyptian children. Eur J Hosp Pharm. (2018). 10.1136/ejhpharm-2018-001707 PMC7223341 32419935

[20] Greenberg RA Kerby G Roosevelt GE. A comparison of oral dexamethasone with oral prednisone in pediatric asthma exacerbations treated in the emergency department. Clin Pediatr. (2008) 47:817–23. 10.1177/0009922808316988 18467673

[21] Paniagua N Lopez R Muñoz N Tames M Mojica E Arana-Arri E. Randomized trial of dexamethasone versus prednisone for children with acute asthma exacerbations. J Pediatr. (2017) 191:190–6.e1. 10.1016/j.jpeds.2017.08.030 29173304

[22] Cooper WO Hickson GB Corticosteroid prescription filling for children covered by medicaid following an emergency department visit or a hospitalization for asthma. Arch Pediatr Adolesc Med. (2001) 155:1111 10.1001/archpedi.155.10.1111 11576005

[23] Scarfone RJ Loiselle JM Wiley JF Decker JM Henretig FM Joffe MD. Nebulized dexamethasone versus oral prednisone in the emergency treatment of asthmatic children. Ann Emerg Med. (1995) 26:480–6. 10.1016/S0196-0644(95)70118-4 7574132

[24] Evans DD Clinton Shedd G. Treating adult asthma exacerbations with a 2-day course of dexamethasone in the emergency department. Adv Emerg Nurs J. (2016) 38:171–6. 10.1097/TME.0000000000000109 27482988

[25] Kravitz J Dominici P Ufberg J Fisher J Giraldo P. Two days of dexamethasone versus 5 days of prednisone in the treatment of acute asthma: a randomized controlled trial. Ann Emerg Med. (2011) 58:200–4. 10.1016/j.annemergmed.2011.01.004 21334098

[26] Rehrer MW Liu B Rodriguez M Lam J Alter HJ A randomized controlled non-inferiority trial of single dose of oral dexamethasone versus 5 days of oral prednisone in acute adult asthma. Ann Emerg Med. (2016) 68:608–13. 10.1016/j.annemergmed.2016.03.017 27117874

[27] Benito-Fernández J Onis-González E Álvarez-Pitti J Capapé-Zache S Vázquez-Ronco MA Mintegi-Raso S. Factors associated with short-term clinical outcomes after acute treatment of asthma in a pediatric emergency department. Pediatr Pulmonol. (2004) 38:123–8. 10.1002/ppul,0.20031 15211695

[28] Go SI Koo DH Kim ST Song HN Kim RB Jang JS. Antiemetic corticosteroid rotation from dexamethasone to methylprednisolone to prevent dexamethasone-induced hiccup in cancer patients treated with chemotherapy: a randomized, single-blind, crossover phase III trial. Oncologist. (2017) 22:1354–61. 10.1634/theoncologist.2017-0129 28687626 PMC5679820

[29] Hames H Seabrook JA Matsui D Rieder MJ Joubert GI. A palatability study of a flavored dexamethasone preparation versus prednisolone liquid in children. Can J Clin Pharmacol. (2008) 15:e95–98. 18245869

